# Comparative validation of LC-MS, micro-bioassay, and ELISA for lincomycin quantification: application to pharmacokinetic analysis in healthy and co-infected porcine models

**DOI:** 10.1080/01652176.2026.2708928

**Published:** 2026-07-25

**Authors:** Syed Al Jawad Sayem, Seung-Jin Lee, Ling Gui, Md. Sekendar Ali, Seung-Chun Park

**Affiliations:** a Laboratory of Veterinary Pharmacokinetics and Pharmacodynamics, Institute for Veterinary Biomedical Science, College of Veterinary Medicine, Kyungpook National University, Daegu, South Korea; b Developmental and Reproductive Toxicology Research Group, Korea Institute of Toxicology, Daejeon, South Korea; c Department of Pharmacy, International Islamic University Chittagong, Kumira, Chittagong, Bangladesh

**Keywords:** Lincomycin, LC-MS, micro-bioassay, ELISA, pharmacokinetics, porcine respiratory disease, PK/PD integration

## Abstract

This study compared liquid chromatography–mass spectrometry (LC-MS), micro-bioassay (MBa), and enzyme-linked immunosorbent assay (ELISA) for quantification of lincomycin (10 mg/kg BW) in porcine serum and evaluated lincomycin pharmacokinetic (PK) data in healthy piglets also co-infected with *Actinobacillus pleuropneumoniae* and *Pasteurella multocida*. All methods satisfied standard bioanalytical validation criteria, including low limits of detection and quantification, acceptable intra-assay imprecision and error, and strong linearity across clinically relevant concentration ranges. Serum concentrations measured by MBa and ELISA showed high correlation with LC-MS in both healthy and infected models (Pearson r ≥ 0.97, low bias on Bland–Altman analysis); they produced comparable PK parameters (Cmax, Tmax, area under the curve [AUC], clearance, and mean residence time), indicating suitability for PK/pharmacodynamic (PD)-based dose optimization. PK analysis demonstrated rapid absorption after intramuscular administration and disease-associated alterations in selected distribution and disposition parameters. AUC and clearance were nearly similar across analytical methods and largely consistent between software platforms (WinNonlin and PKSolver). Thus, lower-cost MBa and ELISA—combined with freely available PKSolver—can generate robust, decision-relevant PK/PD data for lincomycin in swine, providing a practical framework for antimicrobial dose refinement and stewardship in resource-limited veterinary and farm settings.

## 1. Introduction

The global swine industry faces substantial economic challenges due to the high prevalence and impact of infectious respiratory diseases, which frequently require herd-level interventions and impose significant financial burdens on producers (Boeters et al. 2023). Key bacterial pathogens associated with these conditions include *Bordetella bronchiseptica*, *Mycoplasma hyopneumoniae*, *Actinobacillus pleuropneumoniae*, and *Pasteurella multocida*, all of which contribute to severe and prolonged respiratory illness, particularly when opportunistic microorganisms result in co-infections within the porcine respiratory disease complex (Brockmeier et al. 2002). Morbidity rates related with porcine respiratory diseases range from 30% to 70%; reported fatality rates are between 4% and 6%, or potentially higher, in affected farmhouses (Opriessnig et al. 2011).

Lincomycin, a lincosamide antibiotic first isolated in the early 1960s, is widely used in pig farming because of its activity against a broad range of bacterial pathogens, including gram-positive bacteria, anaerobic non–spore-forming bacteria, and certain atypical organisms such as *Mycoplasma* spp. (Leigh 1981; Mrowczynski et al. 2018). Its antibacterial effect is mediated through inhibition of bacterial protein synthesis via binding to the 50S ribosomal subunit. Lincomycin is commonly selected for veterinary use in livestock, particularly to manage infections that are resistant to penicillin (Koutsoumanis et al. 2021). However, the efficacy of lincomycin, similar to other antimicrobials, is increasingly compromised by the rapid emergence of bacterial resistance, which represents a major global public health concern. This growing resistance, together with a decline in new antimicrobial development, highlights the critical importance of optimising existing agents through rigorous pharmacokinetic (PK) and pharmacodynamic (PD) investigations (Albarellos et al. 2012; Bengtsson and Greko 2014).

PK data are essential for defining optimal dosage regimens, dosing schedules, and routes of administration. The integration of PK/PD data is critical for ensuring therapeutic efficacy, minimising adverse effects, and effectively limiting the development of antimicrobial resistance. Key PK parameters, including clearance and bioavailability, directly inform dose determination, together with PK/PD indices such as Cmax/MIC, AUC/MIC, and T > MIC, which characterise antimicrobial killing activity (Reigner and Blesch 2002; Trivedi et al. 2013). In particular, *A. pleuropneumoniae* and *P. multocida* are major swine respiratory pathogens; *A. pleuropneumoniae* causes severe fibrinous haemorrhagic and necrotising pleuropneumonia (Andreasen et al. 2001; Liu et al. 2003; Zimmerman and Karriker, 2019), whereas *P. multocida* acts as an important secondary invader within the porcine respiratory disease complex. An understanding of the comparative PK characteristics of lincomycin in healthy and co-infected animals is crucial for effective disease management (Wilkie et al. 2012; Wallgren et al. 2016).

Accurate determination of serum antibiotic concentrations is essential for reliable PK/PD analysis and subsequent dose optimisation. Several analytical techniques, including high-performance liquid chromatography, liquid chromatography–mass spectrometry (LC-MS), micro-bioassay (MBa), and enzyme-linked immunosorbent assay (ELISA), have been utilised for this purpose (Issa et al. 2007; Yücel et al. 2018). Each method presents distinct strengths and limitations. As gold standard LC-MS—indispensable for precise and selective quantification of antibiotics and their metabolites—is associated with high capital and maintenance costs, operational complexity requiring specialised expertise, and potential sensitivity challenges arising from high levels of endogenous proteins in biological matrices (Hortin and Sviridov 2010; Liu et al. 2014; Cross and Hornshaw 2016). MBa and ELISA represent practical alternatives because they are less complex, more cost-effective, and more accessible to laboratories lacking advanced instrumentation, thereby addressing several limitations inherent to LC-MS–based analysis (Mendez et al. 2005). Accordingly, we selected LC MS, ELISA and MBa as the focus of this comparison because they collectively represent the main analytical approaches currently used for antibiotic quantification: a highly sensitive chromatographic–mass spectrometric method, a widely available kit-based immunoassay, and a low-cost microbiological assay that directly reflects antibacterial activity. This combination was chosen to evaluate whether simpler, more accessible techniques can reliably complement or substitute LC MS for PK applications, particularly in laboratories that lack advanced instrumentation.

In parallel with chromatographic, microbiological and immunoassay-based techniques, voltametric and other electrochemical methods have recently emerged as attractive tools for antibiotic detection in food, environmental and veterinary matrices. These sensor platforms, often based on modified electrodes or nanomaterials, offer rapid analysis, high sensitivity, relatively low cost and the possibility of portable, on-site measurements, and thus are promising complements to conventional analytical techniques (Adane et al. 2024, 2025); however, their application in fully validated serum PK assays in veterinary settings is still limited at present.

Despite the importance of accurate quantification and the known influence of infection on drug PK parameters, a clear gap remains in the current literature. To our knowledge, no comprehensive study has systematically compared the performance of LC-MS, MBa, and ELISA for lincomycin quantification in porcine serum, particularly in healthy pigs versus animals co-infected with *A. pleuropneumoniae* and *P. multocida*. Moreover, a direct comparative PK evaluation of lincomycin in healthy and co-infected piglets has not yet been reported.

Therefore, this study explored two primary objectives. First, we conducted a rigorous methodological comparison of LC-MS, MBa, and ELISA for lincomycin quantification in porcine serum, using Pearson correlation coefficients and Bland–Altman analysis at a 95% confidence level, to identify the most time-efficient and cost-effective technique that could be reliably utilised as an alternative or complementary approach to LC-MS. Establishment of strong correlations between LC-MS and the more accessible MBa or ELISA would substantially facilitate the development of efficient and economically viable antibacterial therapies, particularly in resource-limited settings. The principal intent was not to replace LC-MS, but to provide validated alternative options. Second, differences in lincomycin PK parameters were assessed between healthy piglets and piglets co-infected with *A. pleuropneumoniae* and *P. multocida*, using analytical results generated from the three quantification methods. Additionally, PK parameter estimates obtained with two different software programmes, WinNonlin and PKSolver—a free, menu-driven Microsoft Excel add-in (written in Visual Basic for Applications) for PK and PD analysis—were compared to validate the results and evaluate the feasibility of PKSolver use in local settings such as clinics or farms (Zhang et al. 2010a). Integration of these findings may contribute to reduced development of drug-resistant bacteria and lower mortality rates by enabling effective and timely emergency management of infections.

## 2. Materials and methods

### 2.1. Chemicals and components

Lincomycin (analytical grade) used in this study was obtained from Sigma (St. Louis, MO, USA) and exhibited a purity greater than 98%. The injectable formulation of lincomycin was purchased from Samyang Anipharm (Seoul, Korea). All reagents used in this investigation were of high-performance liquid chromatography grade.

### 2.2. Microbial strains

To establish the microbiological assay, the indicator strain *Micrococcus luteus* KCCM 11236 was obtained from the Korean Culture Centre of Microorganisms and cultured for 18 h at 37 °C in tryptic soy broth (Becton Dickinson Company). The test pathogens *A. pleuropneumoniae* (BA1500394) and *P. multocida* (BA1700127) were supplied by the Animal and Plant Quarantine Agency (Gimcheon, Korea) and were maintained at 37 °C on brain–heart infusion agar (Becton Dickinson Company, NJ, USA) supplemented with 5% sheep blood prior to use in experiments.

### 2.3. Experimental design

The study included 16 piglets aged 5–6 weeks, with a mean body weight of 8.5 ± 1.0 kg. Animals underwent a 1-week acclimatisation period with *ad libitum* access to water and feed. After acclimatisation, piglets were randomly assigned to two groups: a control group comprising eight healthy animals and an experimental group comprising eight pigs subjected to co-infection. The animal experiment was approved by the Animal Ethics Committee of the Petobio Clinical Institute (PTB-2022-IACUC-013-A). A 40-mL bacterial culture suspension was centrifuged at 3,500 rpm for 10 min; the supernatant was then discarded, and the bacterial pellet was resuspended in 40 mL of 0.9% NaCl. To establish the disease model, pigs in the experimental group were intranasally inoculated with 1 mL of a mixed suspension containing 2.0 × 10^9^ colony-forming units (CFU)/mL of both *A. pleuropneumoniae* and *P. multocida*. Throughout the experiment, clinical parameters—including respiratory signs, general appearance, rectal temperature, and other relevant indicators—were monitored according to previously described methods (Halbur et al. 1995; Oliviero et al. 2009).

### 2.4. Bacterial culture conditions and media

The two bacterial strains were streaked onto Mueller–Hinton (MH) agar plates supplemented with 3% defibrinated sheep blood and incubated at 37 °C for 24 h. Two to three individual colonies from freshly prepared plates were inoculated into MH broth supplemented with 3% defibrinated sheep blood. Cultures were incubated at 37 °C in a shaking incubator with a centrifugal force of 773.5 × g until the logarithmic growth phase was reached, as previously described (Oliveira et al. 2003). Immediately before bacterial administration to the porcine subjects, amplified cultures were diluted in 0.9% NaCl (pH 7.4). This procedure yielded a final volume of 5 mL with an approximate concentration of 2 × 10^9^ CFU/mL, which served as the bacterial inoculum.

### 2.5. Polymerase chain reaction assay

Polymerase chain reaction (PCR) assays were performed targeting two genes. The *apxIVA* gene was used as an indicator of *A. pleuropneumoniae* infection, whereas the *kmt1* gene was used for detection of *P. multocida* (Da Costa et al. 2004; Shalaby et al. 2021). A commercial PCR kit (AccuPower®, Bioneer) containing primers specific for *A. pleuropneumoniae* and *P. multocida* was utilised. For PCR testing, samples were collected using cotton-tipped nasal swabs inserted approximately 2 cm into the nasal cavity of pigs. After collection, the swabs were immediately returned to sterile plastic tubes and maintained on ice throughout the sampling period. Upon retrieval, swabs were either stored at −20 °C or immediately processed for PCR analysis. Genomic DNA was extracted according to the method described by Schaller et al. (Schaller et al. 2001). PCR amplification was conducted in accordance with the manufacturer’s instructions, including adherence to the specified thermal cycling conditions.

### 2.6. Blood collection and serum sample processing

At predefined time points (0, 0.25, 0.5, 0.75, 1, 2, 4, 6, 8, 12, 16, and 24 h) after 10 mg/kg BW lincomycin administration, blood samples (2 mL each) were collected from the jugular vein for PK analysis. Serum was separated by centrifugation at 3,000 rpm for 10 min and stored at −70 °C until analysis. Sample preparation for LC-MS analysis was performed as follows. Serum aliquots (250 µL) were thawed at ambient temperature, after which deproteinization was achieved by addition of 2 mL acetonitrile and vortex mixing for 15 s. The mixture was centrifuged at 5,000 × g for 10 min at 4 °C. Subsequently, 2 mL of the supernatant were concentrated using a vacuum centrifuge (Modulspin 31, Hanil Scientific, Korea) at 50 °C. The residue was reconstituted in 200 µL methanol, subjected to ultrasonication for 10 min, and centrifuged at 12,000 rpm for 10 min at 4 °C. Finally, 70 µL of the supernatant was utilised for LC-MS analysis.

### 2.7. Analysis of serum lincomycin concentration

#### 2.7.1. Micro-bioassay

**Figure 1.**
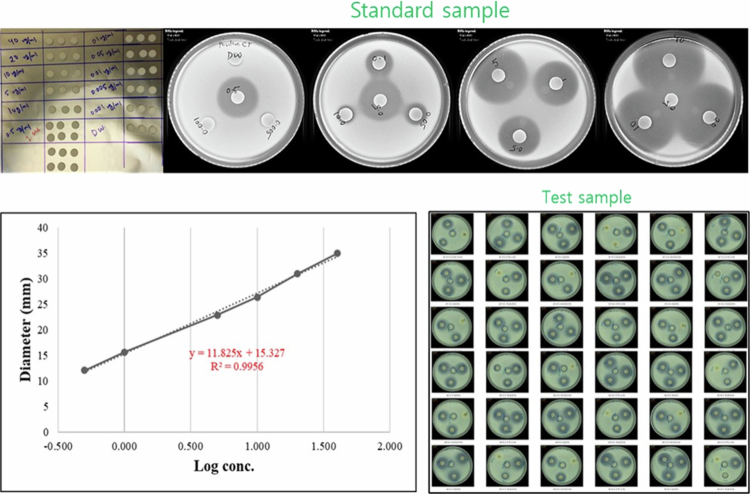
Inhibition diameter (mm) for defining standard curve of microbiological assay.

Serum lincomycin were determined using a microbiological assay in which *M. luteus* KCCM 11236 served as the test bacterium, as shown in Figure 1 (Bennett et al. 1966). The bacterial suspension was prepared after a 24 h incubation in tryptic soy broth. Later, the *M. luteus* KCCM 11236 was spread onto nutrient agar (Becton Dickinson Company, NJ, USA). The bacterial concentration was adjusted to 10^5^ CFU/mL. The agar slurry was promptly poured onto assay plates to form layers approximately 2.2 mm in thickness. After a solidification period of 30 min, paper discs (0.5 cm in diameter) were placed onto the agar surface. The discs were then loaded with serum samples or lincomycin standards covering a concentration range of 0.001-40 µg/mL (60 µL per disk). Plates were incubated at 37 °C for 20 h. Zones of bacterial growth inhibition were measured using a caliper (Mitutoyo, Japan). The procedure was validated according to previously described methods (Strachunskii et al. 1993; Cazedey and Salgado 2011). The assay showed good linearity over 0.05–20 µg/mL, with an r^2^ of 0.99. The limit of detection (LOD) and limit of quantification (LOQ) were both 0.01 µg/mL, corresponding to the lowest calibration standard that consistently produced a measurable inhibition zone distinguishable from the blank (Figure 1).

#### 2.7.2. Enzyme-linked immunosorbent assay (ELISA)

Serum lincomycin concentrations were determined using a commercial ELISA kit obtained from Amsbio (Abingdon, Oxfordshire, UK). A serum aliquot (1 mL) was transferred into a 2-mL microcentrifuge tube and centrifuged at 4,000 rpm for 5 min at ambient temperature. After centrifugation, 0.1 mL of the supernatant was collected and mixed with 0.9 mL of reconstitution buffer supplied with the ELISA kit. The mixture was gently oscillated for 30 s, after which a 50-µL aliquot was withdrawn for analysis in accordance with the manufacturer’s instructions. Optical density was measured at 450 nm using an Epoch microplate reader (BioTek, Winooski, VT, USA). Absorbance percentage was calculated using the equation (A/A_0_) × 100%, where A represents the mean absorbance of the standard or sample and A_0_ represents the mean absorbance of the 0 µg/mL standard. To construct the standard curve, absorbance percentage values were plotted on the y-axis against the log concentration on the x-axis, generating a semi-logarithmic curve. Assay precision was assessed by repeatability and expressed as the relative standard deviation (RSD). According to the manufacturer’s validation data, the assay sensitivity was 0.0002 µg/mL and the practical LOD and LOQ in serum, after sample preparation and dilution, was 0.005 µg/mL. For samples with concentrations above the upper calibration standard, serum was further diluted with assay buffer to bring the measured values within the validated calibration range, and the final lincomycin concentration was obtained by multiplying by the appropriate dilution factor.

#### 2.7.3. Liquid chromatography-mass spectrometry (LC-MS) analysis

LC–MS analysis was performed with minor modifications to a previously reported method (Du et al. 2021). An Agilent 1200 high-performance liquid chromatography system (Agilent Technologies, Santa Clara, CA, USA) was coupled to an Agilent 6140 single-quadrupole mass spectrometer equipped with an electrospray ionisation source operated in positive ion mode. Chromatographic separation was achieved at 35 °C on an Eclipse Plus C18 column (2.1 × 100 mm, 3.5 µm; Agilent Technologies, Santa Clara, CA, USA). The mobile phase consisted of eluent A (5 mmol/L ammonium acetate containing 0.1% formic acid in water) and eluent B (acetonitrile), delivered at a flow rate of 0.3 mL/min, with an injection volume of 4 µL. The gradient programme was initiated at 10% B, increased to 30% over 3 min, then raised to 90% over the next 5 min and held for 2 min, before returning to the initial conditions over 9 min to allow adequate column re-equilibration.​ The mass spectrometer was tuned to maximise analyte signal, with the ion spray voltage set at 5,500 V and the source temperature maintained at 550 °C. Source gas settings (nebulising and drying gases) were adjusted according to the manufacturer’s recommendations to ensure stable spray and optimal sensitivity. The limit of detection was defined as the lowest concentration that produced a signal-to-noise ratio greater than 3 in blank samples spiked with the analyte, and the limit of quantification was defined as the lowest concentration that produced a signal-to-noise ratio greater than 10 under the same conditions.

### 2.8. Comparative PK parameters study

PK analysis was performed using LC-MS to evaluate serum samples obtained from healthy and diseased porcine subjects. Lincomycin PK parameters were estimated using first-order and two-compartment models with WinNonlin 8.3 software (Certara, Wayne, PA, USA) and PKSolver, a freely accessible Microsoft Excel add-in (written in Visual Basic for Applications) for analysis of PK and PD data. Peak concentration (Cmax) and time to peak concentration (Tmax) were determined after intramuscular administration of lincomycin. Additional parameters, including area under the concentration–time curve (AUC), half-life (T1/2), clearance, and mean residence time (MRT), were calculated using the linear trapezoidal method.

### 2.9. Statistical analysis

Statistical studies, with data processing and graphical presentation, were performed using GraphPad Prism 8.0 (GraphPad Software Inc., La Jolla, CA, USA). Concordance among the three analytical methods was assessed using Pearson correlation coefficients and Bland–Altman analysis.

## 3. Results

### 3.1. Clinical factors and target gene finding

**Figure 2.**
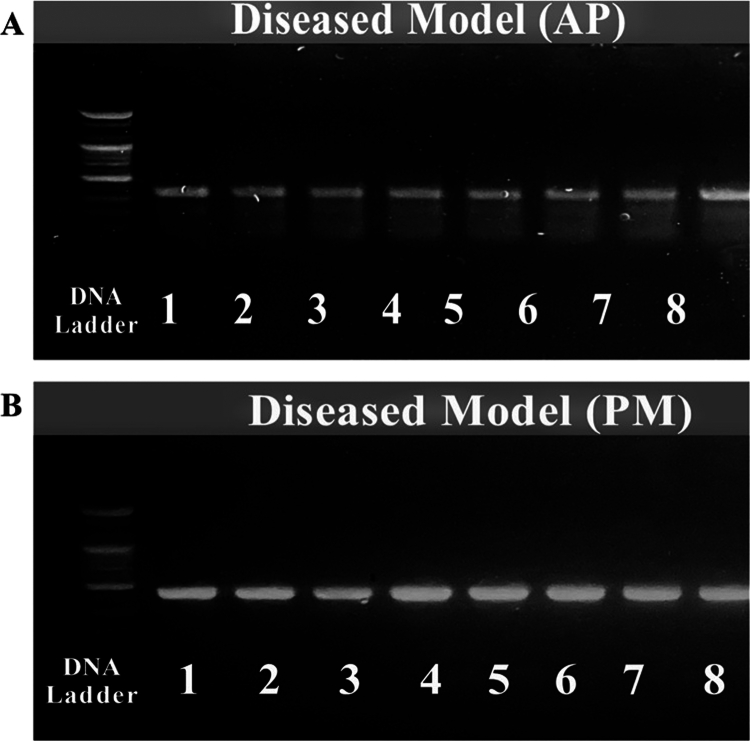
Polymerase chain reaction (PCR) confirmation of *A. pleuropneumoniae* (AP) and *P. multocida* (PM). (A) PCR products amplified with primers targeting the *apxIVA* gene of *A. pleuropneumoniae*. (B) PCR products amplified with primers targeting the *kmt1* gene of *P. multocida*. L: 100 bp DNA ladder.

After successful establishment of the co-infection disease model, clinical manifestations were compared between diseased and healthy animals. Sixteen pigs were evaluated: eight healthy pigs and eight diseased pigs. Among the infected animals, five exhibited overt clinical signs, including reduced activity and laboured breathing. The remaining infected pigs showed lethargy and decreased interaction with their environment relative to uninfected controls. Pigs inoculated with *A. pleuropneumoniae* and *P. multocida* tested positive for the *apxIVA* and *kmt1* genes, which served as PCR targets for *A. pleuropneumoniae* and *P. multocida*, respectively. The amplified fragments measured 377 bp (Figure 2A) and 460 bp (Figure 2B), providing confirmation of infection.

### 3.2. Characteristics of infected pigs

At 10 h after inoculation, pigs in the infection group showed no discernible clinical signs or increases in rectal temperature. Subsequently, early clinical symptoms appeared sequentially, including crouching, dyspnoea, reduced appetite, coughing, and decreased mobility. At approximately 18 h after inoculation, infected pigs developed a mildly elevated rectal temperature and exhibited lethargy. A significantly increased rectal temperature (mean 106 °F, *p* < 0.001 compared with normal rectal temperature) was observed 32 h after inoculation. At this stage, affected pigs showed difficulty supporting body weight on one limb during movement. Additional clinical signs, including joint swelling and mucopurulent nasal discharge, were observed at 44 h after inoculation. By 72 h post-inoculation, two pigs had died. In contrast, all healthy pigs remained clinically normal throughout the experimental period.

### 3.3. Determination of serum lincomycin

#### 3.3.1. Serum concentration by bioassay

**Figure 3.**
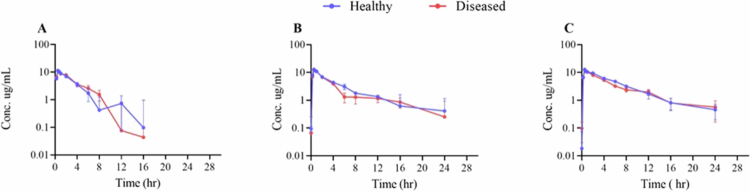
Serum concentration–time profiles after intramuscular administration of lincomycin at a dose of 10 mg/kg bw, measured using (A) MBa, (B) LC-MS, and (C) ELISA. conc., concentration; Lin, lincomycin.

Serum lincomycin concentrations after intramuscular administration are illustrated in Figure 3A. The bioassay demonstrated limits of detection and quantification comprising 0.05 µg/mL for lincomycin in serum. Linearity was observed over the concentration range of 0.05–20 µg/mL, with a high correlation coefficient (R^2^ = 0.99). In Figure 1, inhibition diameters used to construct the standard curve are displayed. In this study, healthy pigs exhibited a peak serum concentration of 10.45 µg/mL, whereas pigs in the diseased model reached a peak concentration of 10.92 µg/mL at Tmax values of 0.78 h and 0.99 h, respectively. As shown in Table 1, the intra-assay evaluation yielded a mean RSD of 3.047% and mean error of 4.550%. These results indicate that the microbiological assay met accepted criteria for quantitative determination of lincomycin in serum, with RSD values near 2% and acceptable error percentages.

**Table 1.** Intra-assay variation in the micro-bioassay (MBa).

Targeted concentration (µg/mL)	Actual mean concentration (µg/mL)	Precision (RSD %)	Intra-assay error (%)
0.05	0.0480	5.101	4%
0.1	0.0950	4.255	5%
0.5	0.532	4.640	6%
1	0.944	4.048	6%
5	4.86	0.987	3%
10	10.3	1.997	3%
20	20.9	0.302	4%
	Mean	3.047%	4.550%

RSD: relative standard deviation.

#### 3.3.2. Serum concentration by LC-MS

The concentration–time profiles of lincomycin are depicted in Figure 3B. Method linearity was evaluated using lincomycin standards with concentrations ranging from 0.025 to 8 µg/mL. The limits of detection and quantification were determined to be 0.01 µg/mL and 0.05 µg/mL, respectively. The Cmax of lincomycin was 12.16 µg/mL in healthy pigs and 11.61 µg/mL in diseased pigs. Tmax was 0.64 h in healthy pigs and 0.72 h in diseased pigs. Based on intra-assay evaluation (Table 2), the mean error percentage was 2.0%, and the mean RSD was 1.466%. The LC-MS assay met accepted criteria for quantitative determination in serum, as indicated by RSD values below 2% and error percentages lower than those observed with the MBa.

**Table 2.** Intra-assay variation in the liquid chromatography–mass spectrometry (LC-MS) method.

Targeted concentration (µg/mL)	Actual mean concentration (µg/mL)	Precision (RSD %)	Intra-assay error (%)
0.025	0.0240	2.373	3%
0.05	0.0480	1.195	3%
0.1	0.0980	0.591	2%
1	0.970	2.728	3%
4	4.11	0.243	3%
8	8.04	1.670	1%
	Mean	1.466%	2%

RSD: relative standard deviation.

#### 3.3.3. Serum concentration by ELISA

Serum lincomycin concentrations measured at different time points using ELISA are presented in Figure 3C. The limits of detection and quantification for lincomycin in serum were both 0.005 µg/mL. In this assay, the healthy group exhibited a peak concentration of 11.62 µg/mL; the diseased group showed a peak concentration of 11.19 µg/mL. Maximum concentrations were achieved at 0.78 h in healthy pigs and 0.77 h in infected pigs (Tmax). According to the intra-assay evaluation (Table 3), the mean error percentage was 2.977%, which was higher than that observed with the other methods, reflecting lower analytical accuracy; the mean RSD was 2.0%. Because the RSD values remained within acceptable limits, ELISA met the criteria for quantitative determination in serum samples.

**Table 3.** Intra-assay variation in the enzyme-linked immunosorbent assay (ELISA).

Targeted concentration (µg/mL)	Actual mean concentration (µg/mL)	Precision (RSD %)	Intra-assay error (%)
0.2	0.197	2.936	2%
0.4	0.407	3.756	2%
0.8	0.773	3.733	3%
1.6	1.58	2.227	1%
3.2	3.24	2.230	1%
	Mean	2%	2.977%

RSD: relative standard deviation.

### 3.4. Association of methods

#### 3.4.1. Correlation of LC-MS vs. Micro-bioassay

**Figure 4.**
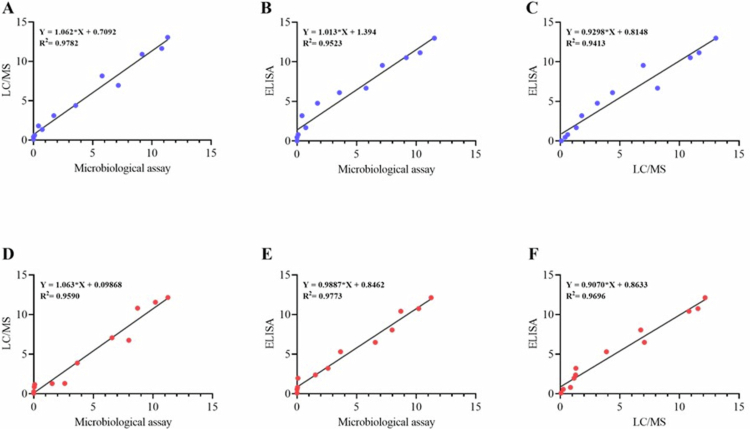
Correlation of serum lincomycin concentrations measured by MBa, ELISA, and LC-MS in healthy and diseased pigs. Correlation plots of concentrations obtained using (A) LC-MS and MBa in healthy pigs, (B) MBa and ELISA in healthy pigs, (C) LC-MS and ELISA in healthy pigs, (D) LC-MS and MBa in diseased pigs, (E) MBa and ELISA in diseased pigs, and (F) LC-MS and ELISA in diseased pigs.

**Figure 5.**
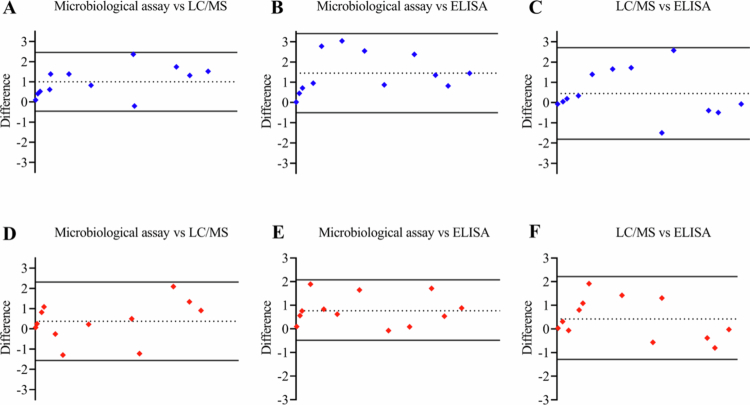
Bland–Altman plots of serum lincomycin concentrations determined using LC-MS, MBa, and ELISA, with confidence interval limits for the mean difference and limits of agreement. Bland–Altman plots of (A) LC-MS vs. MBa in healthy pigs, (B) MBa vs. ELISA in healthy pigs, (C) LC-MS vs. ELISA in healthy pigs, (D) LC-MS vs. MBa in diseased pigs, (E) MBa vs. ELISA in diseased pigs, and (F) LC-MS vs. ELISA in diseased pigs.

Figure 4A illustrates the relationship between serum lincomycin concentrations measured by LC-MS and the MBa in healthy pigs. A Pearson correlation coefficient of 0.9890 (*p* < 0.001) and an estimated slope of 1.062 indicate a strong and statistically significant positive association between the two methods. A similarly strong correlation was observed in diseased piglets, with a Pearson correlation coefficient of 0.9793 (*p* < 0.001) and a slope of 1.063 (Figure 4d). Bland–Altman analysis showed limits of agreement of 2.457 and 0.460 in healthy pigs (Figure 5A). In the disease model, the limits of agreement were 2.313 and −1.560 (Figure 5D). In both groups, the narrow bias range indicated strong concordance between LC-MS and MBa for quantification of serum lincomycin concentrations.

#### 3.4.2. Correlation of Micro-bioassay and ELISA

Figure 4B presents the relationship between serum lincomycin concentrations quantified by the MBa and ELISA. In healthy pigs, a Pearson correlation coefficient of 0.9795 (*p* < 0.001) and slope of 1.013 were observed. In diseased pigs, the Pearson correlation coefficient was 0.9886 (*p* < 0.001) and the slope was 0.9887 (Figure 4E; Table 4). Bland–Altman analysis demonstrated satisfactory agreement between MBa and ELISA for serum lincomycin quantification. The mean bias between the two methods was 1.447 in healthy pigs and 0.797 in diseased pigs at the 95% confidence level. In healthy pigs, the limits of agreement were 3.401 and −0.507 (Figure 5B), whereas in diseased pigs, the limits of agreement were 2.075 and −0.482 (Figure 5E).

**Table 4.** Pearson correlation coefficients and Bland–Altman parameters in healthy and diseased piglets.

Technique	Parameter	Healthy	Diseased
Microbiological assay vs. LC-MS	Pearson coefficient	0.9890	0.9793
	Bias	0.998	0.3766
	Upper LOA	2.457	2.313
	Lower LOA	0.460	−1.560
Microbiological assay vs. ELISA	Pearson coefficient	0.9795	0.9886
	Bias	1.447	0.797
	Upper LOA	3.401	2.075
	Lower LOA	−0.507	−0.482
LC-MS vs. ELISA	Pearson coefficient	0.9702	0.9847
	Bias	0.449	0.421
	Upper LOA	2.711	2.126
	Lower LOA	−1.813	−1.286

LOA: limit of agreement.

#### 3.4.3. Correlation of LC-MS and ELISA

Figure 4C shows a clear positive association between serum lincomycin concentrations measured by LC-MS and ELISA in healthy pigs. The Pearson correlation coefficient of 0.9702 (*p* < 0.001) indicated a strong correlation, and the slope of 0.9298 suggested consistency between the two methods across the concentration range. A similar association was observed in diseased pigs, with a Pearson correlation coefficient of 0.9847 (*p* < 0.001) and a slope of 0.9070 (Figure 4F). Bland–Altman analysis was performed to assess agreement between LC-MS and ELISA. In healthy pigs, the mean bias was 0.449, with limits of agreement of 2.771 and −1.813 (Figure 5C). Conversely, the mean bias in diseased pigs, was 0.421, with limits of agreement of 2.126 and −1.286 at the 95% confidence level (Figure 5F). These findings indicate good agreement between LC-MS and ELISA for quantification of serum lincomycin concentrations.

#### 3.4.4. PK parameter comparison

**Figure 6.**
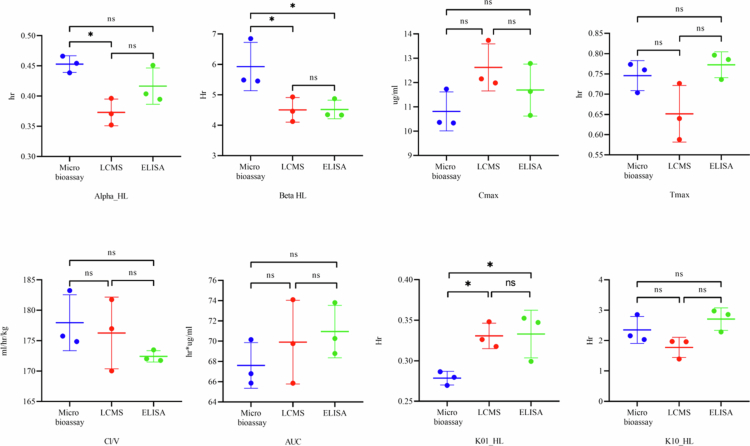
Comparison of key PK parameters obtained using MBa, LC-MS, and ELISA. Alpha_HL, half-life of the distribution phase; Beta_HL, half-life of the elimination phase; K01_HL, distribution rate constant; K10_HL, elimination rate constant; Cmax, maximum observed concentration after dosing; Tmax, time to reach maximum observed concentration, CL/F, clearance corrected for bioavailability; AUC, area under the concentration–time curve.

**Figure 7.**
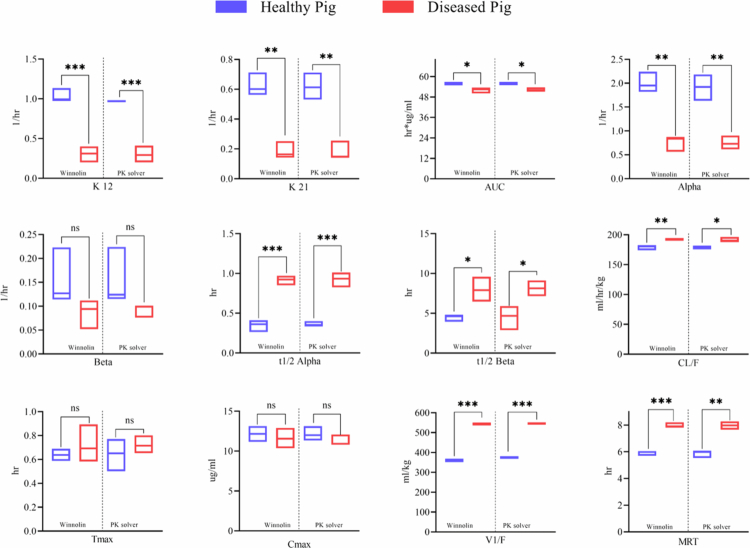
Comparison of PK parameters between diseased and healthy pigs calculated using WinNonlin and PKSolver. K12, rate constant from central to peripheral compartment; K21, rate constant from peripheral to central compartment; t1/2 Alpha, half-life of the distribution phase; t1/2 Beta, half-life of the elimination phase; V1/F, volume of distribution corrected for bioavailability; CL/F, clearance corrected for bioavailability; Tmax, time to reach maximum observed concentration; Cmax, maximum observed concentration after dosing; AUC, area under the concentration–time curve; MRT, mean residence time.

Key PK parameters derived from the lincomycin concentration–time profiles using the three analytical methods and calculated with WinNonlin software are presented in Figure 6. Overall, PK parameters showed no statistically significant differences among MBa, LC-MS, and ELISA. For Alpha_HL, a modest but statistically significant difference was observed between MBa and LC-MS (*p* < 0.05). For Beta_HL, MBa showed a slight but significant difference compared with LC-MS and ELISA (*p* < 0.05). The distribution rate constant (K01_HL) also showed a substantial difference among MBa, LC-MS, and ELISA (*p* < 0.05). No meaningful differences were observed for the remaining parameters, indicating their suitability for equivalent dose calculations. Minor variations among selected PK parameters were not considered to affect estimation of comparable doses when PK/PD integration was performed (Figure 7).

## 4. Discussion

Effective management of the porcine respiratory disease complex relies on timely and appropriate antibiotic administration to control secondary bacterial infections (Odland and Edler, 2022). However, the increasing threat of antimicrobial resistance in both veterinary and human medicine necessitates a shift from empirical dosing practices toward regimens optimised through integrated PK/PD approaches. PK/PD modelling and integration are widely recognised as essential tools for designing rational dosage regimens that maximise therapeutic efficacy while minimising the selection and amplification of resistant bacterial populations (Ahmad et al. 2016). Most contemporary PK studies of antibacterial agents, including those used for respiratory infections in pigs, rely on high-performance analytical platforms such as LC-MS or LC-MS/MS for drug quantification. Although analytically superior (Lu et al. 2022), these platforms are expensive to acquire and maintain and require specialised technical expertise and infrastructure. Such requirements create practical barriers for economically constrained universities, small research centres, diagnostic laboratories, and farm-level facilities, particularly in low- and middle-income countries. A clear methodological gap has existed regarding whether simpler, lower-cost techniques can reliably replace or complement LC-MS for generation of PK profiles suitable for PK/PD integration and dose optimisation (Dafale et al. 2016). The present study helps address that gap by directly comparing three quantification methods—LC-MS, MBa, and ELISA—for lincomycin in porcine serum and demonstrating that the less expensive and technically simpler methods (MBa and ELISA) show strong correlation and acceptable agreement with LC-MS. In addition to their lower cost, MBa and ELISA therefore provide analytically reliable and operationally practical alternatives to LC‑MS for generating PK/PD‑relevant lincomycin exposure data in routine veterinary settings. By confirming that MBa and ELISA can yield PK parameters, including Cmax, Tmax, AUC, and half-lives, with sufficient accuracy for dose calculation, this work provides a practical, time- and cost-effective analytical framework tailored to resource-limited research and farm environments.

A second substantive knowledge gap concerns the impact of clinically relevant co-infections on lincomycin PK parameters in pigs. Although lincomycin has been evaluated in several species and under healthy conditions, detailed PK characterisation in pigs concurrently infected with *A. pleuropneumoniae* and *P. multocida*—two major bacterial contributors to the porcine respiratory disease complex—has been lacking. Previous studies have largely focused on single-pathogen models, healthy animals, or residue and tissue depletion, leaving uncertainty as to whether disease-associated physiological changes (e.g., inflammation, altered perfusion, fever) meaningfully influence lincomycin disposition in the clinical settings where it is most frequently administered.

In this study, LC-MS, MBa, and ELISA were systematically compared for lincomycin quantification in porcine serum; lincomycin PK data were evaluated in healthy and co-infected piglets. All three methods demonstrated excellent analytical performance. The MBa showed a linear range of 0.05–20 µg/mL, with limits of detection and quantification of 0.05 µg/mL, an average intra-assay RSD of 3.047%, and a mean error of 4.550%. LC-MS exhibited a linear range of 0.025–8 µg/mL, with a limit of detection of 0.01 µg/mL, a limit of quantification of 0.05 µg/mL, an RSD of 1.466%, and an average error of 2.0%. ELISA achieved the lowest limit of detection and limit of quantification (0.005 µg/mL), with a mean error of 2.977% and an RSD of 2.0%. These performance metrics fall within widely accepted bioanalytical criteria for quantitative antibiotic assays and are comparable to, or better than, values reported for lincomycin and other antimicrobials in previous PK and therapeutic monitoring studies (Tiwari and Tiwari 2010; Fda and Cder 2022).

Strong agreement among the three methods was further supported by correlation and concordance analyses. In healthy pigs, Pearson correlation coefficients were 0.9890 for LC-MS versus MBa, 0.9795 for MBa versus ELISA, and 0.9702 for LC-MS versus ELISA, with similarly high coefficients evident in diseased pigs. Regression slopes were close to unity, indicating that all three techniques generated comparable concentration estimates across the measured range (Martin Bland and Altman 1986). Bland–Altman analysis showed low bias values, confirming good interchangeability of the methods for PK applications. Similar levels of concordance have been reported when microbiological or immunoassay-based approaches were carefully calibrated against chromatographic–mass spectrometric methods for antibiotic quantification (Mansournia et al. 2020).

The PK profiles obtained with each analytical method were numerically consistent and demonstrated rapid absorption following intramuscular administration. Using the MBa, mean Cmax values were 10.45 µg/mL in healthy pigs and 10.92 µg/mL in diseased pigs; corresponding Tmax values were 0.78 h and 0.99 h. LC-MS produced slightly higher Cmax estimates of 12.16 µg/mL in healthy pigs and 11.61 µg/mL in diseased pigs, with Tmax values of 0.64 h and 0.72 h, respectively. ELISA indicated Cmax values of 11.62 µg/mL in healthy animals and 11.19 µg/mL in diseased animals, occurring at Tmax values of 0.78 h and 0.77 h. These peak concentrations and absorption times are consistent with previous reports indicating that lincomycin reaches maximal plasma concentrations within approximately 30–60 min after intramuscular administration and maintains bacteriostatic serum levels for up to 12 h, depending on dose and species (Nielsen and Gyrd-Hansen 1998; Ding et al. 2008). Minor statistical differences observed for selected parameters did not materially affect AUC, clearance, or mean residence time and are therefore unlikely to influence PK/PD-based dose optimisation.

PK parameters calculated using WinNonlin and PKSolver exhibited highly consistent patterns of statistical significance when healthy and diseased pigs were compared, despite small numerical differences attributable to the underlying computational algorithms. Because infection and inflammation can alter drug clearance, distribution and protein binding, PK profiles obtained in healthy animals alone may not reflect exposure in clinically diseased pigs. By characterising lincomycin PK in both healthy and co-infected piglets, we aimed to capture these disease-related changes so that any PK/PD-based dosing recommendations are directly relevant to the target clinical population (Martinez et al. 2020). Parameters such as K12, K21, t1/2 Alpha, clearance corrected for bioavailability, V1/F, and MRT showed significant decreases or increases in diseased animals across both software platforms, whereas t1/2 Beta, Tmax, and Cmax consistently showed no significant differences. This concordance indicates that the direction and interpretation of disease-associated PK changes remain robust, regardless of analytical software. Because PK-based decision-making primarily depends on whether physiological or pathological conditions meaningfully alter drug disposition, rather than on minor differences in absolute parameter values, agreement in significance outcomes is particularly important (Tyson et al. 2020). These findings collectively support use of PKSolver as a scientifically valid and resource-efficient alternative to WinNonlin, especially in low-income academic, clinical, and veterinary research settings where high licensing costs limit access to commercial PK software. Previous evaluations have indicated that PKSolver yields compartmental and noncompartmental PK parameters comparable to those generated by professional platforms such as WinNonlin and Scientist, supporting its suitability as a free analytical tool for routine PK research (Zhang et al. 2010b).

A key translational aspect of this work concerns the economic and infrastructural implications of exclusive reliance on LC-MS. LC-MS/MS platforms are widely regarded as high-cost technologies, with clinical implementation requiring substantial capital investment in the mass spectrometer, liquid chromatography system, gas supply, and facility modifications. For example, a detailed cost analysis of a clinical LC-MS/MS installation revealed that the system, including the mass spectrometer, LC components, and necessary infrastructure, represents a substantial initial investment, reflecting the high expense associated with establishing such analytical capability (Grebe and Singh 2011; Shushan 2008). Additionally, laboratories must account for ongoing operational costs, including annual service contracts and recurring expenses associated with maintenance and consumables such as columns, gases, and solvents. Operation requires specialised technical personnel, along with stable environmental and power conditions, which are frequently unavailable in veterinary laboratories in low- and middle-income countries. In contrast, MBa can be implemented using standard incubators, agar plates, and basic microbiological equipment, whereas ELISA requires only a microplate reader and relatively simple sample preparation. These lower technological requirements, together with the demonstrated numerical agreement (correlation coefficients ≥0.97, low bias, and RSD ≤ 3.1%), make MBa and ELISA particularly suitable for low-resource settings where advanced LC-MS infrastructure is neither accessible nor sustainable.

Finally, the numerical consistency of PK parameters calculated using WinNonlin and PKSolver indicates that high-cost commercial software is not strictly required for reliable PK/PD interpretation. PKSolver, a free Excel add-in, reproduced compartmental and noncompartmental parameters that closely matched those obtained with WinNonlin when applied to this dataset, corroborating previous validation studies that showed strong agreement between the two platforms across multiple drug classes. When combined with low-cost analytical approaches such as MBa and ELISA, this level of computational accessibility enables locally conducted PK studies, dose optimisation, and resistance-mitigation strategies in regions where both LC-MS instrumentation and commercial PK software are prohibitively expensive. Collectively, the numerical and methodological evidence presented here supports a scalable framework in which resource-appropriate analytical and modelling tools can be leveraged to improve lincomycin use and antimicrobial stewardship in the global swine industry, particularly in low- and middle-income countries.

## 5. Conclusion

This study demonstrates that lincomycin PK parameters in pigs can be accurately and reliably characterised without exclusive reliance on LC-MS or costly commercial PK software. Quantification by MBa and ELISA satisfied accepted bioanalytical validation criteria, showed very high correlation and close agreement with LC-MS, and generated comparable PK parameters and exposure estimates suitable for PK/PD-based dose optimisation. In addition, MBa and ELISA provide practical advantages such as simpler workflows, faster processing of multiple samples and reduced dependence on highly specialised LC-MS infrastructure, making them realistic options for routine PK work in laboratories with limited resources. Across healthy and co-infected animals, all three analytical methods indicated rapid absorption and disease-related alterations in selected PK parameters, without meaningful differences in AUC, clearance, or mean residence time. Furthermore, compartmental analyses conducted via WinNonlin and the freely available PKSolver programme yielded nearly identical patterns of statistically significant disease effects. The present findings address a critical methodological gap by establishing that lower-cost analytical techniques, when combined with freely accessible PK software, can deliver robust PK/PD information, supporting locally driven dose refinement and antimicrobial stewardship for lincomycin in swine, particularly in economically constrained veterinary and farm settings.

## Data Availability

The data obtained in the present study are available within the article and supplementary materials.

## References

[cit0001] Adane WD , Chandravanshi BS , Tessema M . 2025. Multi-elemental nanocomposite electrochemical sensor for the simultaneous determination of azithromycin and enrofloxacin residues in food and water samples. Microchem J. 210:113042. 10.1016/J.MICROC.2025.113042

[cit0002] Adane WD , Chandravanshi BS , Chebude Y , Tessema M . 2024. A novel electrochemical sensor (Au-Ag-ANCCs/r-GO/poly(L-histidine)/GCE) for the simultaneous determination of vancomycin and ceftriaxone residues in chicken meat, fish, and milk samples. Chem Eng J. 497:154808. 10.1016/J.CEJ.2024.154808

[cit0003] Ahmad I , Huang L , Hao H , Sanders P , Yuan Z . 2016. Application of pk/pd modeling in veterinary field: dose optimization and drug resistance prediction. BioMed Res Int. 2016:5465678. 10.1155/2016/5465678 26989688 PMC4771886

[cit0004] Albarellos GA , Montoya L , Denamiel GAA , Velo MC , Landoni MF . 2012. Pharmacokinetics and bone tissue concentrations of lincomycin following intravenous and intramuscular administrations to cats. Vet Pharmacol Ther. 35(6):534–540. 10.1111/j.1365-2885.2011.01355.x 22132730

[cit0005] Andreasen M , Mousing J , Thomsen LK . 2001. No overall relationship between average daily weight gain and the serological response to *mycoplasma hyopneumoniae* and *actinobacillus pleuropneumoniae* in eight chronically infected danish swine herds. Prev Vet Med. 49(1–2):19–28. 10.1016/S0167-5877(01)00174-X 11267685

[cit0006] Bengtsson B , Greko C . 2014. Antibiotic resistance—consequences for animal health, welfare, and food production. Ups J Med Sci. 119(2):96–102. 10.3109/03009734.2014.901445 24678738 PMC4034566

[cit0007] Bennett JV , Brodie JL , Benner EJ , Kirby WMM . 1966. Simplified, accurate method for antibiotic assay of clinical specimens. Appl Microbiol. 14(2):170–177. 10.1128/AM.14.2.170-177.1966 4959982 PMC546645

[cit0008] Boeters M , Garcia-Morante B , van Schaik G , Segalés J , Rushton J , Steeneveld W . 2023. The economic impact of endemic respiratory disease in pigs and related interventions - a systematic review. Porcine Health Manag. 9(1):45. 10.1186/S40813-023-00342-W/TABLES/4 37848972 PMC10583309

[cit0009] Brockmeier SL , Halbur PG , Thacker EL . 2002. Porcine Respiratory Disease Complex - Polymicrobial Diseases - NCBI Bookshelf. https://www.ncbi.nlm.nih.gov/books/NBK2481/

[cit0010] Cazedey ECL , Salgado HRN . 2011. Development and validation of a microbiological agar assay for determination of orbifloxacin in pharmaceutical preparations. Pharmaceutics. 3(3):572–581. 10.3390/PHARMACEUTICS3030572 24310597 PMC3857083

[cit0011] Cross TG , Hornshaw MP . 2016. Can LC and LC-MS ever replace immunoassays? J Appl Bioanal. 2(4):108–116. 10.17145/JAB.16.015

[cit0012] Da Costa MM et al. 2004. Evaluation of PCR based on gene apxIVA associated with 16S rDNA sequencing for the identification of *actinobacillus pleuropneumoniae* and related species. Curr Microbiol. 48(3):189–195. 10.1007/S00284-003-4162-X 15057463

[cit0013] Dafale NA , Semwal UP , Rajput RK , Singh GN . 2016. Selection of appropriate analytical tools to determine the potency and bioactivity of antibiotics and antibiotic resistance. J Pharm Anal. 6(4):207–213. 10.1016/J.JPHA.2016.05.006 29403984 PMC5762606

[cit0014] Ding HZ , Yang GX , Huang XH , Chen ZL , Zeng ZL . 2008. Pharmacokinetics of difloxacin in pigs and broilers following intravenous, intramuscular, and oral single-dose applications. J Vet Pharmacol Ther. 31(3):200–204. 10.1111/j.1365-2885.2008.00951.x 18471140

[cit0015] Du L et al. 2021. Establishment and validation of the LC-MS/MS method for the determination of lincomycin in human blood: application to an allergy case in forensic science. J Forensic Leg Med. 77:102094. 10.1016/J.JFLM.2020.102094 33383379

[cit0016] Fda , Cder . 2022. M10 bioanalytical method validation and study sample analysis; Guidance for Industry. https://www.fda.gov/vaccines-blood-biologics/guidance-compliance-regulatory-information-biologics/biologics-guidances

[cit0017] Grebe SKG , Singh RJ . 2011. LC-MS/MS in the clinical laboratory – where to from here? Clin Biochem Rev. 32(1):5. https://pmc.ncbi.nlm.nih.gov/articles/PMC3052391/. 21451775 PMC3052391

[cit0018] Halbur PG et al. 1995. Comparison of the pathogenicity of two US porcine reproductive and respiratory syndrome virus isolates with that of the *lelystad virus* . Vet Pathol. 32(6):648–660. 10.1177/030098589503200606 8592800

[cit0019] Hortin GL , Sviridov D . 2010. The dynamic range problem in the analysis of the plasma proteome. J Proteomics. 73(3):629–636. 10.1016/j.jprot.2009.07.001 19619681

[cit0020] Issa MM , Nejem RM , El-Abadla NS , El-Naby MK , Roshdy AA , Kheiralla ZA . 2007. Effects of paracetamol on the pharmacokinetics of ciprofloxacin in plasma using a microbiological assay. Clin Drug Investig. 27(7):463–467. 10.2165/00044011-200727070-00003 17563126

[cit0021] Koutsoumanis K et al. 2021. Maximum levels of cross‐contamination for 24 antimicrobial active substances in non‐target feed. Part 5: lincosamides: lincomycin. EFSA J. 19(10):e06856. 10.2903/J.EFSA.2021.6856 34729085 PMC8546522

[cit0022] Leigh DA . 1981. Antibacterial activity and pharmacokinetics of clindamycin. J Antimicrob Chemother. 7(suppl_A):3–9. 10.1093/JAC/7.SUPPL_A.3 7019193

[cit0023] Shushan B , Zonderman J . 2008. Liquid Chromatography Coupled with Tandem Mass Spectrometry for Clinical Applications. LCGC International. https://www.chromatographyonline.com/view/liquid-chromatography-coupled-tandem-mass-spectrometry-clinical-applications-0

[cit0024] Liu J , Fung KF , Chen Z , Zeng Z , Zhang J . 2003. Pharmacokinetics of florfenicol in healthy pigs and in pigs experimentally infected with *actinobacillus pleuropneumoniae* . Antimicrob Agents Chemother. 47(2):820–823. 10.1128/AAC.47.2.820-823.2003 12543702 PMC151723

[cit0025] Liu G , Zhao Y , Angeles A , Hamuro LL , Arnold ME , Shen JX . 2014. A novel and cost effective method of removing excess albumin from Plasma/Serum samples and its impacts on LC-MS/MS bioanalysis of therapeutic proteins. Anal Chem. 86(16):8336–8343. 10.1021/AC501837T 25083595

[cit0026] Lu W et al. 2022. An LC-MS/MS method for the simultaneous determination of 18 antibacterial drugs in human plasma and its application in therapeutic drug monitoring. Front Pharmacol. 13:1044234. 10.3389/FPHAR.2022.1044234 36425576 PMC9679284

[cit0027] Mansournia MA , Waters R , Nazemipour M , Bland M , Altman DG . 2020. Bland-altman methods for comparing methods of measurement and response to criticisms. Glob Epidemiol. 3:100045. 10.1016/J.GLOEPI.2020.100045 37635723 PMC10446118

[cit0028] Martin Bland J , Altman DG . 1986. Statistical methods for assessing agreement between two methods of clinical measurement. Lancet. 327(8476):307–310. 10.1016/S0140-6736(86)90837-8 2868172

[cit0029] Martinez MN , Greene J , Kenna L , Kissell L , Kuhn M . 2020. The impact of infection and inflammation on drug metabolism, active transport, and systemic drug concentrations in veterinary species. Drug Metab Dispos. 48(8):631–644. 10.1124/dmd.120.090704 32503881

[cit0030] Mendez ASL , Weisheimer V , Oppe TP , Steppe M , Schapoval EES . 2005. Microbiological assay for the determination of meropenem in pharmaceutical dosage form. J Pharm Biomed Anal. 37(4):649–653. 10.1016/j.jpba.2004.11.030 15797784

[cit0031] Mrowczynski OD , Langan ST , Rizk EB . 2018. Intra-cerebrospinal fluid antibiotics to treat central nervous system infections: a review and update. Clin Neurol Neurosurg. 170:140–158. 10.1016/j.clineuro.2018.05.007 29800828

[cit0032] Nielsen P , Gyrd-Hansen N . 1998. Bioavailability of spiramycin and lincomycin after oral administration to fed and fasted pigs. J Vet Pharmacol Ther. 21(4):251–256. 10.1046/J.1365-2885.1998.00131.X 9731946

[cit0033] Odland CA , Edler R , Noyes NR , Dee SA , Nerem J , Davies PR . 2022. Evaluation of the impact of antimicrobial use protocols in porcine reproductive and respiratory syndrome virus-infected swine on phenotypic antimicrobial resistance patterns. Appl Environ Microbiol. 88(1):e0097021. 10.1128/AEM.00970-21;WGROUP:STRING:PUBLICATION 34644164 PMC8752131

[cit0034] Oliveira S , Blackall PJ , Pijoan C . 2003. Characterization of the diversity of *haemophilus parasuis* field isolates by use of serotyping and genotyping. Am J Vet Res. 64(4):435–442. 10.2460/AJVR.2003.64.435 12693533

[cit0035] Oliviero C , Kokkonen T , Heinonen M , Sankari S , Peltoniemi O . 2009. Feeding sows with high fibre diet around farrowing and early lactation: impact on intestinal activity, energy balance related parameters and litter performance. Res Vet Sci. 86(2):314–319. 10.1016/J.RVSC.2008.07.007 18725160

[cit0036] Opriessnig T , Giménez-Lirola LG , Halbur PG . 2011. Polymicrobial respiratory disease in pigs. Anim Health Res Rev. 12(2):133–148. 10.1017/S1466252311000120 22152290

[cit0037] Reigner BG , Blesch K . 2002. Estimating the starting dose for entry into humans: principles and practice. Eur J Clin Pharmacol. 57(12):835–845. 10.1007/S00228-001-0405-6 11936701

[cit0038] Schaller A et al. 2001. Identification and detection of *actinobacillus pleuropneumoniae* by PCR based on the gene apxIVA. Vet Microbiol. 79(1):47–62. 10.1016/S0378-1135(00)00345-X 11230928

[cit0039] Shalaby AG , Bakry NR , El-Demerdash AS . 2021. Virulence attitude estimation of *pasteurella multocida* isolates in embryonated chicken eggs. Arch Microbiol. 203(10):6153–6162. 10.1007/S00203-021-02579-X 34554268

[cit0040] Strachunskii LS et al. 1993. Evaluation of the bioavailability of preparations subjected to hepato-intestinal circulation: lincomycin. Pharm Chem J. 27(12):814–818. 10.1007/BF00780570/METRICS

[cit0041] Tiwari G , Tiwari R . 2010. Bioanalytical method validation: an updated review. Pharm Methods. 1(1):25. 10.4103/2229-4708.72226 23781413 PMC3658022

[cit0042] Trivedi A , Lee RE , Meibohm B . 2013. Applications of pharmacometrics in the clinical development and pharmacotherapy of anti-infectives. Expert Rev Clin Pharmacol. 6(2):159–170. 10.1586/ECP.13.6 23473593 PMC3809903

[cit0043] Tyson RJ et al. 2020. Precision dosing priority criteria: drug, disease, and patient population variables. Front Pharmacol. 11:420. 10.3389/FPHAR.2020.00420 32390828 PMC7188913

[cit0044] Wallgren P , Nörregård E , Molander B , Persson M , Ehlorsson CJ . 2016. Serological patterns of *actinobacillus pleuropneumoniae, mycoplasma hyopneumoniae, pasteurella multocida* and *streptococcus suis* in pig herds affected by pleuritis. Acta Vet Scand. 58(1):71. 10.1186/S13028-016-0252-1 27716292 PMC5050615

[cit0045] Wilkie IW , Harper M , Boyce JD , Adler B . 2012. *Pasteurella multocida*: diseases and pathogenesis. Curr Top Microbiol Immunol. 361:1–22. 10.1007/82_2012_216 22643916

[cit0046] Yücel K , Abuşoǧlu S , Ünlü A . 2018. Comparison of immunoassay and liquid chromatography-tandem mass spectrometry methods in the measurement of serum androstenedione levels. Clin Lab. 64(1):69–75. 10.7754/CLIN.LAB.2017.170612 29479885

[cit0047] Zhang Y , Huo M , Zhou J , Xie S . 2010a. PKSolver: an add-in program for pharmacokinetic and pharmacodynamic data analysis in microsoft excel. Comput Methods Programs Biomed. 99(3):306–314. 10.1016/j.cmpb.2010.01.007 20176408

[cit0048] Zhang Y , Huo M , Zhou J , Xie S . 2010b. PKSolver: an add-in program for pharmacokinetic and pharmacodynamic data analysis in microsoft excel. Comput Methods Programs Biomed. 99(3):306–314. 10.1016/J.CMPB.2010.01.007 20176408

[cit0049] Zimmerman JJ , Karriker LA , Ramirez A , Schwartz KJ , Stevenson GW , Zhang J . 2019. Diseases of Swine. 11th ed. Wiley-Blackwell: Hoboken (NJ). 10.1002/9781119350927

